# Supplementary material for: Virulence Genes and In Vitro Antibiotic Profile of Photobacterium damselae Strains, Isolated from Fish Reared in Greek Aquaculture Facilities
Source: Animals (Basel). 2022 Nov 14;12(22):3133. doi: 10.3390/ani12223133 (PMC9687077; doi:10.3390/ani12223133)
Supplement: Supplementary file 1 [file animals-12-03133-s001.zip › animals-2025482-supplementary.pdf]

>120

GTAATGGCTCACCAAGGCAACGATCCCTAGCTGGTCTGAGAGGATGATCAGCCACAC
TGGAACTGAGACACGGTCCAGACTCCTACGGGAGGCAGCAGTGGGGAATATTGCACA
ATGGGGGAAACCCTGATGCAGCCATGCCGCGTGTGTGAAGAAGGCCTTCGGGTTGTA
AAGCACTTTCAGTAGGGAGGAAGGTAGTGTAGTTAACACCTGCACTATTTGACGTTA
CCTACAGAAGAAGCACCGGCTAACTCCGTGCCAGCAGCCGCGGTAATACGGAGGGTG
CGAGCGTTAATCGGAATTACTGGGCGTAAAGCGCATGCAGGCGGCCTGTTAAGTCAG
ATGTGAAAGCCCGGGGCTTAACCTCGGAATTGCATTTGAAACTGGCAGGCTAGAGTC
TTGTAGAGGGGGGTAGAATTTCAGGTGTAGCGGTGAAATGCGTAGAGATCTGAAGGA
ATACCAGTGGCGAAGGCGGCCCCCTGGACAAAGACTGACGCTCAGATGCGAAAGCGT
GGGGAGCAAACAGGATTAGATACCCTGGTAGTCCACGCCGTAAACGATGTCTACTTG
GAGGTTGTGGCCTTGAGCCGTGGCTTTCGGAGCTAACGCGTTAAGTAGACCGCCTGG
GGAGTACGGTCGCAAGATTAAAACTCAAATGAATTGACGGGGGCCCGCACAAGCGGT
GGAGCATGTGGTTTAATTCGATGCAACGCGAAGAACCTTACCTACTCTTGACATCCA
GAGAAGCTTGAAGAGATTCGAGTGTGCCTTCGGGAACTCTGAGACAGGTGCTGCATG
GCTGTCGTCAGCTCGTGTTGTGAAATGTTGGGTTAAGTCCCGCAACGAGCGCAACCCT
TATCCTTGTTTGCCAGCACTTCGGGTGGGAACTCCAGGGAGACTGCCGGTGATAAAC
CGGAGGAAGGTGGGGACGACGTCAAGTCATCATGGCCCTTACGAGTAGGGCTACACA
CGTGCTACAATGGC

>214

GTAATGGCTCACCAAGGCAACGATCCCTAGCTGGTCTGAGAGGATGATCAGCCACAC
TGGAACTGAGACACGGTCCAGACTCCTACGGGAGGCAGCAGTGGGGAATATTGCACA
ATGGGGGAAACCCTGATGCAGCCATGCCGCGTGTGTGAAGAAGGCCTTCGGGTTGTA
AAGCACTTTCAGTAGGGAGGAAGGTAGTGTAGTTAACACCTGCACTATTTGACGTTA
CCTACAGAAGAAGCACCGGCTAACTCCGTGCCAGCAGCCGCGGTAATACGGAGGGTG
CGAGCGTTAATCGGAATTACTGGGCGTAAAGCGCATGCAGGCGGCCTGTTAAGTCAG
ATGTGAAAGCCCGGGGCTTAACCTCGGAATTGCATTTGAAACTGGCAGGCTAGAGTC
TTGTAGAGGGGGGTAGAATTTCAGGTGTAGCGGTGAAATGCGTAGAGATCTGAAGGA
ATACCAGTGGCGAAGGCGGCCCCCTGGACAAAGACTGACGCTCAGATGCGAAAGCGT
GGGGAGCAAACAGGATTAGATACCCTGGTAGTCCACGCCGTAAACGATGTCTACTTG
GAGGTTGTGGCCTTGAGCCGTGGCTTTCGGAGCTAACGCGTTAAGTAGACCGCCTGG
GGAGTACGGTCGCAAGATTAAAACTCAAATGAATTGACGGGGGCCCGCACAAGCGGT
GGAGCATGTGGTTTAATTCGATGCAACGCGAAGAACCTTACCTACTCTTGACATCCA
GAGAAGCTTGAAGAGATTCGAGTGTGCCTTCGGGAACTCTGAGACAGGTGCTGCATG
GCTGTCGTCAGCTCGTGTTGTGAAATGTTGGGTTAAGTCCCGCAACGAGCGCAACCCT
TATCCTTGTTTGCCAGCACTTCGGGTGGGAACTCCAGGGAGACTGCCGGTGATAAAC
CGGAGGAAGGTGGGGACGACGTCAAGTCATCATGGCCCTTACGAGTAGGGCTACACA
CGTGCTACAATGGC

>222

GTAATGGCTCACCAAGGCAACGATCCCTAGCTGGTCTGAGAGGATGATCAGCCACAC
TGGAACTGAGACACGGTCCAGACTCCTACGGGAGGCAGCAGTGGGGAATATTGCACA
ATGGGGGAAACCCTGATGCAGCCATGCCGCGTGTGTGAAGAAGGCCTTCGGGTTGTA
AAGCACTTTCAGTAGGGAGGAAGGTAGTGTAGTTAACACCTGCACTATTTGACGTTA
CCTACAGAAGAAGCACCGGCTAACTCCGTGCCAGCAGCCGCGGTAATACGGAGGGTG

CGAGCGTTAATCGGAATTACTGGGCGTAAAGCGCATGCAGGCGGCCTGTTAAGTCAG
ATGTGAAAGCCCGGGGCTTAACCTCGGAATTGCATTTGAAACTGGCAGGCTAGAGTC
TTGTAGAGGGGGGTAGAATTTCAGGTGTAGCGGTGAAATGCGTAGAGATCTGAAGGA
ATACCAGTGGCGAAGGCGGCCCCCTGGACAAAGACTGACGCTCAGATGCGAAAGCGT
GGGGAGCAAACAGGATTAGATACCCTGGTAGTCCACGCCGTAAACGATGTCTACTTG
GAGGTTGTGGCCTTGAGCCGTGGCTTTCGGAGCTAACGCGTTAAGTAGACCGCCTGG
GGAGTACGGTCGCAAGATTAAAACTCAAATGAATTGACGGGGGCCCGCACAAGCGGT
GGAGCATGTGGTTTAATTCGATGCAACGCGAAGAACCTTACCTACTCTTGACATCCA
GAGAAGCTTGAAGAGATTCGAGTGTGCCTTCGGGAACTCTGAGACAGGTGCTGCATG
GCTGTCGTCAGCTCGTGTTGTGAAATGTTGGGTTAAGTCCCGCAACGAGCGCAACCCT
TATCCTTGTTTGCCAGCACTTCGGGTGGGAACTCCAGGGAGACTGCCGGTGATAAAC
CGGAGGAAGGTGGGGACGACGTCAAGTCATCATGGCCCTTACGAGTAGGGCTACACA
CGTGCTACAATGGC

>228

GTAATGGCTCACCAAGGCAACGATCCCTAGCTGGTCTGAGAGGATGATCAGCCACAC
TGGAACTGAGACACGGTCCAGACTCCTACGGGAGGCAGCAGTGGGGAATATTGCACA
ATGGGGGAAACCCTGATGCAGCCATGCCGCGTGTGTGAAGAAGGCCTTCGGGTTGTA
AAGCACTTTCAGTAGGGAGGAAGGTAGTGTAGTTAACACCTGCACTATTTGACGTTA
CCTACAGAAGAAGCACCGGCTAACTCCGTGCCAGCAGCCGCGGTAATACGGAGGGTG
CGAGCGTTAATCGGAATTACTGGGCGTAAAGCGCATGCAGGCGGCCTGTTAAGTCAG
ATGTGAAAGCCCGGGGCTTAACCTCGGAATTGCATTTGAAACTGGCAGGCTAGAGTC
TTGTAGAGGGGGGTAGAATTTCAGGTGTAGCGGTGAAATGCGTAGAGATCTGAAGGA
ATACCAGTGGCGAAGGCGGCCCCCTGGACAAAGACTGACGCTCAGATGCGAAAGCGT
GGGGAGCAAACAGGATTAGATACCCTGGTAGTCCACGCCGTAAACGATGTCTACTTG
GAGGTTGTGGCCTTGAGCCGTGGCTTTCGGAGCTAACGCGTTAAGTAGACCGCCTGG
GGAGTACGGTCGCAAGATTAAAACTCAAATGAATTGACGGGGGCCCGCACAAGCGGT
GGAGCATGTGGTTTAATTCGATGCAACGCGAAGAACCTTACCTACTCTTGACATCCA
GAGAAGCTTGAAGAGATTCGAGTGTGCCTTCGGGAACTCTGAGACAGGTGCTGCATG
GCTGTCGTCAGCTCGTGTTGTGAAATGTTGGGTTAAGTCCCGCAACGAGCGCAACCCT
TATCCTTGTTTGCCAGCACTTCGGGTGGGAACTCCAGGGAGACTGCCGGTGATAAAC
CGGAGGAAGGTGGGGACGACGTCAAGTCATCATGGCCCTTACGAGTAGGGCTACACA
CGTGCTACAATGGC

>354

GTAATGGCTCACCAAGGCAACGATCCCTAGCTGGTCTGAGAGGATGATCAGCCACAC
TGGAACTGAGACACGGTCCAGACTCCTACGGGAGGCAGCAGTGGGGAATATTGCACA
ATGGGGGAAACCCTGATGCAGCCATGCCGCGTGTGTGAAGAAGGCCTTCGGGTTGTA
AAGCACTTTCAGTAGGGAGGAAGGTAGTGTAGTTAACACCTGCACTATTTGACGTTA
CCTACAGAAGAAGCACCGGCTAACTCCGTGCCAGCAGCCGCGGTAATACGGAGGGTG
CGAGCGTTAATCGGAATTACTGGGCGTAAAGCGCATGCAGGCGGCCTGTTAAGTCAG
ATGTGAAAGCCCGGGGCTTAACCTCGGAATTGCATTTGAAACTGGCAGGCTAGAGTC
TTGTAGAGGGGGGTAGAATTTCAGGTGTAGCGGTGAAATGCGTAGAGATCTGAAGGA
ATACCAGTGGCGAAGGCGGCCCCCTGGACAAAGACTGACGCTCAGATGCGAAAGCGT
GGGGAGCAAACAGGATTAGATACCCTGGTAGTCCACGCCGTAAACGATGTCTACTTG
GAGGTTGTGGCCTTGAGCCGTGGCTTTCGGAGCTAACGCGTTAAGTAGACCGCCTGG
GGAGTACGGTCGCAAGATTAAAACTCAAATGAATTGACGGGGGCCCGCACAAGCGGT

GGAGCATGTGGTTTAATTCGATGCAACGCGAAGAACCTTACCTACTCTTGACATCCA
GAGAAGCTTGAAGAGATTCGAGTGTGCCTTCGGGAACTCTGAGACAGGTGCTGCATG
GCTGTCGTCAGCTCGTGTTGTGAAATGTTGGGTTAAGTCCCGCAACGAGCGCAACCCT
TATCCTTGTTTGCCAGCACTTCGGGTGGGAACTCCAGGGAGACTGCCGGTGATAAAC
CGGAGGAAGGTGGGGACGACGTCAAGTCATCATGGCCCTTACGAGTAGGGCTACACA
CGTGCTACAATGGC

>355

GTAATGGCTCACCAAGGCAACGATCCCTAGCTGGTCTGAGAGGATGATCAGCCACAC
TGGAACTGAGACACGGTCCAGACTCCTACGGGAGGCAGCAGTGGGGAATATTGCACA
ATGGGGGAAACCCTGATGCAGCCATGCCGCGTGTGTGAAGAAGGCCTTCGGGTTGTA
AAGCACTTTCAGTAGGGAGGAAGGTAGTGTAGTTAACACCTGCACTATTTGACGTTA
CCTACAGAAGAAGCACCGGCTAACTCCGTGCCAGCAGCCGCGGTAATACGGAGGGTG
CGAGCGTTAATCGGAATTACTGGGCGTAAAGCGCATGCAGGCGGCCTGTTAAGTCAG
ATGTGAAAGCCCGGGGCTTAACCTCGGAATTGCATTTGAAACTGGCAGGCTAGAGTC
TTGTAGAGGGGGGTAGAATTTCAGGTGTAGCGGTGAAATGCGTAGAGATCTGAAGGA
ATACCAGTGGCGAAGGCGGCCCCCTGGACAAAGACTGACGCTCAGATGCGAAAGCGT
GGGGAGCAAACAGGATTAGATACCCTGGTAGTCCACGCCGTAAACGATGTCTACTTG
GAGGTTGTGGCCTTGAGCCGTGGCTTTCGGAGCTAACGCGTTAAGTAGACCGCCTGG
GGAGTACGGTCGCAAGATTAAAACTCAAATGAATTGACGGGGGCCCGCACAAGCGGT
GGAGCATGTGGTTTAATTCGATGCAACGCGAAGAACCTTACCTACTCTTGACATCCA
GAGAAGCTTGAAGAGATTCGAGTGTGCCTTCGGGAACTCTGAGACAGGTGCTGCATG
GCTGTCGTCAGCTCGTGTTGTGAAATGTTGGGTTAAGTCCCGCAACGAGCGCAACCCT
TATCCTTGTTTGCCAGCACTTCGGGTGGGAACTCCAGGGAGACTGCCGGTGATAAAC
CGGAGGAAGGTGGGGACGACGTCAAGTCATCATGGCCCTTACGAGTAGGGCTACACA
CGTGCTACAATGGC

>367

GTAATGGCTCACCAAGGCAACGATCCCTAGCTGGTCTGAGAGGATGATCAGCCACAC
TGGAACTGAGACACGGTCCAGACTCCTACGGGAGGCAGCAGTGGGGAATATTGCACA
ATGGGGGAAACCCTGATGCAGCCATGCCGCGTGTGTGAAGAAGGCCTTCGGGTTGTA
AAGCACTTTCAGTAGGGAGGAAGGTAGTGTAGTTAACACCTGCATTATTTGACGTTA
CCTACAGAAGAAGCACCGGCTAACTCCGTGCCAGCAGCCGCGGTAATACGGAGGGTG
CGAGCGTTAATCGGAATTACTGGGCGTAAAGCGCATGCAGGCGGCCTGTTAAGTCAG
ATGTGAAAGCCCGGGGCTTAACCTCGGAATTGCATTTGAAACTGGCAGGCTAGAGTC
TTGTAGAGGGGGGTAGAATTTCAGGTGTAGCGGTGAAATGCGTAGAGATCTGAAGGA
ATACCAGTGGCGAAGGCGGCCCCCTGGACAAAGACTGACGCTCAGATGCGAAAGCGT
GGGGAGCAAACAGGATTAGATACCCTGGTAGTCCACGCCGTAAACGATGTCTACTTG
GAGGTTGTGGCCTTGAGCCGTGGCTTTCGGAGCTAACGCGTTAAGTAGACCGCCTGG
GGAGTACGGTCGCAAGATTAAAACTCAAATGAATTGACGGGGGCCCGCACAAGCGGT
GGAGCATGTGGTTTAATTCGATGCAACGCGAAGAACCTTACCTACTCTTGACATCCA
GAGAAGCTTGAAGAGATTCGAGTGTGCCTTCGGGAACTCTGAGACAGGTGCTGCATG
GCTGTCGTCAGCTCGTGTTGTGAAATGTTGGGTTAAGTCCCGCAACGAGCGCAACCCT
TATCCTTGTTTGCCAGCACTTCGGGTGGGAACTCCAGGGAGACTGCCGGTGATAAAC
CGGAGGAAGGTGGGGACGACGTCAAGTCATCATGGCCCTTACGAGTAGGGCTACACA
CGTGCTACAATGGC

>371

GTAATGGCTCACCAAGGCAACGATCCCTAGCTGGTCTGAGAGGATGATCAGCCACAC
TGGAACTGAGACACGGTCCAGACTCCTACGGGAGGCAGCAGTGGGGAATATTGCACA
ATGGGGGAAACCCTGATGCAGCCATGCCGCGTGTGTGAAGAAGGCCTTCGGGTTGTA
AAGCACTTTCAGTAGGGAGGAAGGTAGTGTAGTTAACACCTGCACTATTTGACGTTA
CCTACAGAAGAAGCACCGGCTAACTCCGTGCCAGCAGCCGCGGTAATACGGAGGGTG
CGAGCGTTAATCGGAATTACTGGGCGTAAAGCGCATGCAGGCGGCCTGTTAAGTCAG
ATGTGAAAGCCCGGGGCTTAACCTCGGAATTGCATTTGAAACTGGCAGGCTAGAGTC
TTGTAGAGGGGGGTAGAATTTCAGGTGTAGCGGTGAAATGCGTAGAGATCTGAAGGA
ATACCAGTGGCGAAGGCGGCCCCCTGGACAAAGACTGACGCTCAGATGCGAAAGCGT
GGGGAGCAAACAGGATTAGATACCCTGGTAGTCCACGCCGTAAACGATGTCTACTTG
GAGGTTGTGGCCTTGAGCCGTGGCTTTCGGAGCTAACGCGTTAAGTAGACCGCCTGG
GGAGTACGGTCGCAAGATTAAAACTCAAATGAATTGACGGGGGCCCGCACAAGCGGT
GGAGCATGTGGTTTAATTCGATGCAACGCGAAGAACCTTACCTACTCTTGACATCCA
GAGAAGCTTGAAGAGATTCGAGTGTGCCTTCGGGAACTCTGAGACAGGTGCTGCATG
GCTGTCGTCAGCTCGTGTTGTGAAATGTTGGGTTAAGTCCCGCAACGAGCGCAACCCT
TATCCTTGTTTGCCAGCACTTCGGGTGGGAACTCCAGGGAGACTGCCGGTGATAAAC
CGGAGGAAGGTGGGGACGACGTCAAGTCATCATGGCCCTTACGAGTAGGGCTACACA
CGTGCTACAATGGC

>399

GTAATGGCTCACCAAGGCAACGATCCCTAGCTGGTCTGAGAGGATGATCAGCCACAC
TGGAACTGAGACACGGTCCAGACTCCTACGGGAGGCAGCAGTGGGGAATATTGCACA
ATGGGGGAAACCCTGATGCAGCCATGCCGCGTGTGTGAAGAAGGCCTTCGGGTTGTA
AAGCACTTTCAGTAGGGAGGAAGGTAGTGTAGTTAACACCTGCACTATTTGACGTTA
CCTACAGAAGAAGCACCGGCTAACTCCGTGCCAGCAGCCGCGGTAATACGGAGGGTG
CGAGCGTTAATCGGAATTACTGGGCGTAAAGCGCATGCAGGCGGCCTGTTAAGTCAG
ATGTGAAAGCCCGGGGCTTAACCTCGGAATTGCATTTGAAACTGGCAGGCTAGAGTC
TTGTAGAGGGGGGTAGAATTTCAGGTGTAGCGGTGAAATGCGTAGAGATCTGAAGGA
ATACCAGTGGCGAAGGCGGCCCCCTGGACAAAGACTGACGCTCAGATGCGAAAGCGT
GGGGAGCAAACAGGATTAGATACCCTGGTAGTCCACGCCGTAAACGATGTCTACTTG
GAGGTTGTGGCCTTGAGCCGTGGCTTTCGGAGCTAACGCGTTAAGTAGACCGCCTGG
GGAGTACGGTCGCAAGATTAAAACTCAAATGAATTGACGGGGGCCCGCACAAGCGGT
GGAGCATGTGGTTTAATTCGATGCAACGCGAAGAACCTTACCTACTCTTGACATCCA
GAGAAGCTTGAAGAGATTCGAGTGTGCCTTCGGGAACTCTGAGACAGGTGCTGCATG
GCTGTCGTCAGCTCGTGTTGTGAAATGTTGGGTTAAGTCCCGCAACGAGCGCAACCCT
TATCCTTGTTTGCCAGCACTTCGGGTGGGAACTCCAGGGAGACTGCCGGTGATAAAC
CGGAGGAAGGTGGGGACGACGTCAAGTCATCATGGCCCTTACGAGTAGGGCTACACA
CGTGCTACAATGGC

>400

GTAATGGCTCACCAAGGCAACGATCCCTAGCTGGTCTGAGAGGATGATCAGCCACAC
TGGAACTGAGACACGGTCCAGACTCCTACGGGAGGCAGCAGTGGGGAATATTGCACA
ATGGGGGAAACCCTGATGCAGCCATGCCGCGTGTGTGAAGAAGGCCTTCGGGTTGTA
AAGCACTTTCAGTAGGGAGGAAGGTAGTGTAGTTAACACCTGCACTATTTGACGTTA
CCTACAGAAGAAGCACCGGCTAACTCCGTGCCAGCAGCCGCGGTAATACGGAGGGTG

CGAGCGTTAATCGGAATTACTGGGCGTAAAGCGCATGCAGGCGGCCTGTTAAGTCAG
ATGTGAAAGCCCGGGGCTTAACCTCGGAATTGCATTTGAAACTGGCAGGCTAGAGTC
TTGTAGAGGGGGGTAGAATTTCAGGTGTAGCGGTGAAATGCGTAGAGATCTGAAGGA
ATACCAGTGGCGAAGGCGGCCCCCTGGACAAAGACTGACGCTCAGATGCGAAAGCGT
GGGGAGCAAACAGGATTAGATACCCTGGTAGTCCACGCCGTAAACGATGTCTACTTG
GAGGTTGTGGCCTTGAGCCGTGGCTTTCGGAGCTAACGCGTTAAGTAGACCGCCTGG
GGAGTACGGTCGCAAGATTAAAACTCAAATGAATTGACGGGGGCCCGCACAAGCGGT
GGAGCATGTGGTTTAATTCGATGCAACGCGAAGAACCTTACCTACTCTTGACATCCA
GAGAAGCTTGAAGAGATTCGAGTGTGCCTTCGGGAACTCTGAGACAGGTGCTGCATG
GCTGTCGTCAGCTCGTGTTGTGAAATGTTGGGTTAAGTCCCGCAACGAGCGCAACCCT
TATCCTTGTTTGCCAGCACTTCGGGTGGGAACTCCAGGGAGACTGCCGGTGATAAAC
CGGAGGAAGGTGGGGACGACGTCAAGTCATCATGGCCCTTACGAGTAGGGCTACACA
CGTGCTACAATGGC

>405

GTAATGGCTCACCAAGGCAACGATCCCTAGCTGGTCTGAGAGGATGATCAGCCACAC
TGGAACTGAGACACGGTCCAGACTCCTACGGGAGGCAGCAGTGGGGAATATTGCACA
ATGGGGGAAACCCTGATGCAGCCATGCCGCGTGTGTGAAGAAGGCCTTCGGGTTGTA
AAGCACTTTCAGTAGGGAGGAAGGTAGTGTAGTTAACACCTGCACTATTTGACGTTA
CCTACAGAAGAAGCACCGGCTAACTCCGTGCCAGCAGCCGCGGTAATACGGAGGGTG
CGAGCGTTAATCGGAATTACTGGGCGTAAAGCGCATGCAGGCGGCCTGTTAAGTCAG
ATGTGAAAGCCCGGGGCTTAACCTCGGAATTGCATTTGAAACTGGCAGGCTAGAGTC
TTGTAGAGGGGGGTAGAATTTCAGGTGTAGCGGTGAAATGCGTAGAGATCTGAAGGA
ATACCAGTGGCGAAGGCGGCCCCCTGGACAAAGACTGACGCTCAGATGCGAAAGCGT
GGGGAGCAAACAGGATTAGATACCCTGGTAGTCCACGCCGTAAACGATGTCTACTTG
GAGGTTGTGGCCTTGAGCCGTGGCTTTCGGAGCTAACGCGTTAAGTAGACCGCCTGG
GGAGTACGGTCGCAAGATTAAAACTCAAATGAATTGACGGGGGCCCGCACAAGCGGT
GGAGCATGTGGTTTAATTCGATGCAACGCGAAGAACCTTACCTACTCTTGACATCCA
GAGAAGCTTGAAGAGATTCGAGTGTGCCTTCGGGAACTCTGAGACAGGTGCTGCATG
GCTGTCGTCAGCTCGTGTTGTGAAATGTTGGGTTAAGTCCCGCAACGAGCGCAACCCT
TATCCTTGTTTGCCAGCACTTCGGGTGGGAACTCCAGGGAGACTGCCGGTGATAAAC
CGGAGGAAGGTGGGGACGACGTCAAGTCATCATGGCCCTTACGAGTAGGGCTACACA
CGTGCTACAATGGC

>406

GTAATGGCTCACCAAGGCAACGATCCCTAGCTGGTCTGAGAGGATGATCAGCCACAC
TGGAACTGAGACACGGTCCAGACTCCTACGGGAGGCAGCAGTGGGGAATATTGCACA
ATGGGGGAAACCCTGATGCAGCCATGCCGCGTGTGTGAAGAAGGCCTTCGGGTTGTA
AAGCACTTTCAGTAGGGAGGAAGGTAGTGTAGTTAACACCTGCACTATTTGACGTTA
CCTACAGAAGAAGCACCGGCTAACTCCGTGCCAGCAGCCGCGGTAATACGGAGGGTG
CGAGCGTTAATCGGAATTACTGGGCGTAAAGCGCATGCAGGCGGCCTGTTAAGTCAG
ATGTGAAAGCCCGGGGCTTAACCTCGGAATTGCATTTGAAACTGGCAGGCTAGAGTC
TTGTAGAGGGGGGTAGAATTTCAGGTGTAGCGGTGAAATGCGTAGAGATCTGAAGGA
ATACCAGTGGCGAAGGCGGCCCCCTGGACAAAGACTGACGCTCAGATGCGAAAGCGT
GGGGAGCAAACAGGATTAGATACCCTGGTAGTCCACGCCGTAAACGATGTCTACTTG
GAGGTTGTGGCCTTGAGCCGTGGCTTTCGGAGCTAACGCGTTAAGTAGACCGCCTGG
GGAGTACGGTCGCAAGATTAAAACTCAAATGAATTGACGGGGGCCCGCACAAGCGGT

GGAGCATGTGGTTTAATTCGATGCAACGCGAAGAACCTTACCTACTCTTGACATCCA
GAGAAGCTTGAAGAGATTCGAGTGTGCCTTCGGGAACTCTGAGACAGGTGCTGCATG
GCTGTCGTCAGCTCGTGTTGTGAAATGTTGGGTTAAGTCCCGCAACGAGCGCAACCCT
TATCCTTGTTTGCCAGCACTTCGGGTGGGAACTCCAGGGAGACTGCCGGTGATAAAC
CGGAGGAAGGTGGGGACGACGTCAAGTCATCATGGCCCTTACGAGTAGGGCTACACA
CGTGCTACAATGGC

>407

GTAATGGCTCACCAAGGCAACGATCCCTAGCTGGTCTGAGAGGATGATCAGCCACAC
TGGAACTGAGACACGGTCCAGACTCCTACGGGAGGCAGCAGTGGGGAATATTGCACA
ATGGGGGAAACCCTGATGCAGCCATGCCGCGTGTGTGAAGAAGGCCTTCGGGTTGTA
AAGCACTTTCAGTAGGGAGGAAGGTAGTGTAGTTAACACCTGCACTATTTGACGTTA
CCTACAGAAGAAGCACCGGCTAACTCCGTGCCAGCAGCCGCGGTAATACGGAGGGTG
CGAGCGTTAATCGGAATTACTGGGCGTAAAGCGCATGCAGGCGGCCTGTTAAGTCAG
ATGTGAAAGCCCGGGGCTTAACCTCGGAATTGCATTTGAAACTGGCAGGCTAGAGTC
TTGTAGAGGGGGGTAGAATTTCAGGTGTAGCGGTGAAATGCGTAGAGATCTGAAGGA
ATACCAGTGGCGAAGGCGGCCCCCTGGACAAAGACTGACGCTCAGATGCGAAAGCGT
GGGGAGCAAACAGGATTAGATACCCTGGTAGTCCACGCCGTAAACGATGTCTACTTG
GAGGTTGTGGCCTTGAGCCGTGGCTTTCGGAGCTAACGCGTTAAGTAGACCGCCTGG
GGAGTACGGTCGCAAGATTAAAACTCAAATGAATTGACGGGGGCCCGCACAAGCGGT
GGAGCATGTGGTTTAATTCGATGCAACGCGAAGAACCTTACCTACTCTTGACATCCA
GAGAAGCTTGAAGAGATTCGAGTGTGCCTTCGGGAACTCTGAGACAGGTGCTGCATG
GCTGTCGTCAGCTCGTGTTGTGAAATGTTGGGTTAAGTCCCGCAACGAGCGCAACCCT
TATCCTTGTTTGCCAGCACTTCGGGTGGGAACTCCAGGGAGACTGCCGGTGATAAAC
CGGAGGAAGGTGGGGACGACGTCAAGTCATCATGGCCCTTACGAGTAGGGCTACACA
CGTGCTACAATGGC

>415

GTAATGGCTCACCAAGGCAACGATCCCTAGCTGGTCTGAGAGGATGATCAGCCACAC
TGGAACTGAGACACGGTCCAGACTCCTACGGGAGGCAGCAGTGGGGAATATTGCACA
ATGGGGGAAACCCTGATGCAGCCATGCCGCGTGTGTGAAGAAGGCCTTCGGGTTGTA
AAGCACTTTCAGTAGGGAGGAAGGTAGTGTAGTTAACACCTGCACTATTTGACGTTA
CCTACAGAAGAAGCACCGGCTAACTCCGTGCCAGCAGCCGCGGTAATACGGAGGGTG
CGAGCGTTAATCGGAATTACTGGGCGTAAAGCGCATGCAGGCGGCCTGTTAAGTCAG
ATGTGAAAGCCCGGGGCTTAACCTCGGAATTGCATTTGAAACTGGCAGGCTAGAGTC
TTGTAGAGGGGGGTAGAATTTCAGGTGTAGCGGTGAAATGCGTAGAGATCTGAAGGA
ATACCAGTGGCGAAGGCGGCCCCCTGGACAAAGACTGACGCTCAGATGCGAAAGCGT
GGGGAGCAAACAGGATTAGATACCCTGGTAGTCCACGCCGTAAACGATGTCTACTTG
GAGGTTGTGGCCTTGAGCCGTGGCTTTCGGAGCTAACGCGTTAAGTAGACCGCCTGG
GGAGTACGGTCGCAAGATTAAAACTCAAATGAATTGACGGGGGCCCGCACAAGCGGT
GGAGCATGTGGTTTAATTCGATGCAACGCGAAGAACCTTACCTACTCTTGACATCCA
GAGAAGCTTGAAGAGATTCGAGTGTGCCTTCGGGAACTCTGAGACAGGTGCTGCATG
GCTGTCGTCAGCTCGTGTTGTGAAATGTTGGGTTAAGTCCCGCAACGAGCGCAACCCT
TATCCTTGTTTGCCAGCACTTCGGGTGGGAACTCCAGGGAGACTGCCGGTGATAAAC
CGGAGGAAGGTGGGGACGACGTCAAGTCATCATGGCCCTTACGAGTAGGGCTACACA
CGTGCTACAATGGC

>417

GTAATGGCTCACCAAGGCAACGATCCCTAGCTGGTCTGAGAGGATGATCAGCCACAC
TGGAACTGAGACACGGTCCAGACTCCTACGGGAGGCAGCAGTGGGGAATATTGCACA
ATGGGGGAAACCCTGATGCAGCCATGCCGCGTGTGTGAAGAAGGCCTTCGGGTTGTA
AAGCACTTTCAGTAGGGAGGAAGGTAGTGTAGTTAACACCTGCACTATTTGACGTTA
CCTACAGAAGAAGCACCGGCTAACTCCGTGCCAGCAGCCGCGGTAATACGGAGGGTG
CGAGCGTTAATCGGAATTACTGGGCGTAAAGCGCATGCAGGCGGCCTGTTAAGTCAG
ATGTGAAAGCCCGGGGCTTAACCTCGGAATTGCATTTGAAACTGGCAGGCTAGAGTC
TTGTAGAGGGGGGTAGAATTTCAGGTGTAGCGGTGAAATGCGTAGAGATCTGAAGGA
ATACCAGTGGCGAAGGCGGCCCCCTGGACAAAGACTGACGCTCAGATGCGAAAGCGT
GGGGAGCAAACAGGATTAGATACCCTGGTAGTCCACGCCGTAAACGATGTCTACTTG
GAGGTTGTGGCCTTGAGCCGTGGCTTTCGGAGCTAACGCGTTAAGTAGACCGCCTGG
GGAGTACGGTCGCAAGATTAAAACTCAAATGAATTGACGGGGGCCCGCACAAGCGGT
GGAGCATGTGGTTTAATTCGATGCAACGCGAAGAACCTTACCTACTCTTGACATCCA
GAGAAGCTTGAAGAGATTCGAGTGTGCCTTCGGGAACTCTGAGACAGGTGCTGCATG
GCTGTCGTCAGCTCGTGTTGTGAAATGTTGGGTTAAGTCCCGCAACGAGCGCAACCCT
TATCCTTGTTTGCCAGCACTTCGGGTGGGAACTCCAGGGAGACTGCCGGTGATAAAC
CGGAGGAAGGTGGGGACGACGTCAAGTCATCATGGCCCTTACGAGTAGGGCTACACA
CGTGCTACAATGGC
